# Data on thermal and hydrolytic stability of both domiphen bromide and para-bromodomiphen bromide

**DOI:** 10.1016/j.dib.2018.08.152

**Published:** 2018-08-31

**Authors:** Laura Fumagalli, Alessandra Moretto, Ettore Gilardoni, Claudia Picozzi, Giulio Vistoli, Marina Carini

**Affiliations:** a Dipartimento di Scienze Farmaceutiche, Università degli Studi di Milano, via Mangiagalli 25, I-20133, Milano, Italy; b Dipartimento di Scienze per gli Alimenti, la Nutrizione e l’Ambiente, Università degli Studi di Milano, Via Celoria 2, Milano, Italy

## Abstract

In this data article, HPLC analyses were applied to investigate the hydrolytic and thermal stability of domiphen bromide a FDA approved OTC ingredients endowed with antimicrobial activity. The data obtained by stressing domiphen bromide in acid, base and thermal conditions enlarge the research article published by Fumagalli et al. [2]. The chromatograms herein presented reveal that domiphen bromide is stable under acidic and thermal stress while the treatment with base yield to a by-product. The *para*-bromo derivative, *p*-bromodomiphen bromide shows the same behavior under the up mentioned stressed conditions.

**Specifications table**

**Table:** 

Subject area	Chemistry
More specific subject area	Biopharmaceutical Analysis
Type of data	Figures
How data was acquired	Elite_La Chrom HPLC (VWR/HITACHI, Milan, Italy/Tokyo, Japan) apparatus equipped with a L-2130 high pressure quaternary gradient delivery system, a L-2455 diode array detector (DAD), a L-2300 column oven and a L-2200 autosampler. The separation was achieved on a XBridgeTM column (4.6 mm × 150 mm, 5 μm) (Waters).
Data format	Analyzed.
Experimental factors	100 mg of domiphen bromide/*p*-bromodomiphen bromide dissolved in 2 mL of 1 M NaOH at 80 °C for 48 h.
	100 mg of domiphen bromide/ *p*-bromodomiphen bromide bromide dissolved in 2 mL of 2 M HCl at 80 °C for 48 h.
	100 mg of domiphen bromide/ *p*-bromodomiphen bromide in 2 mL of HPLC-grade water at 100 °C for 4 days.
	100 mg of domiphen bromide/ *p*-bromodomiphen bromide dry at 100 °C for 4 days.
Experimental features	HPLC analyses; chromatograms extracted at 220 nm.
Data source location	University of Milan, Dep. Of Pharmaceutical Sciences, 20133, Milan, Italy
Data accessibility	All data are included in the figures here reported.
Related research article	Stressed degradation studies of domiphen bromide by LC-ESI-MS/MS identify a novel promising antimicrobial agent.

**Value of the data**

● HPLC analyses of domiphen bromide and of *p*-bromo substituted analogue under acidic, basic and thermal stressed conditions better profile its chemical stability.
● The knowledge of the stability of both domiphen bromide and *p*-bromo substituted analogue improves their application in research or production field.
● The data obtained can be used to improve the manufacturing process strategies of different preparations containing domiphen bromide.

## 1 Data

In order to investigate the stability of both domiphen bromide and p-bromodomiphen bromide HPLC analyses of their samples stressed under thermal, acidic and basic conditions were carried out.

**Fig. 1:**
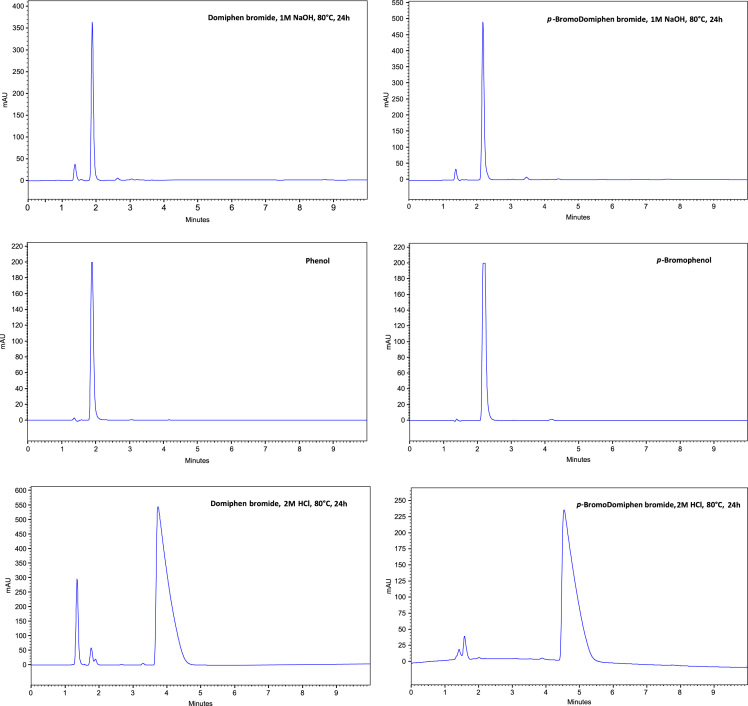
HPLC analyses of domiphen bromide and p-bromodomiphen bromide stressed under hydrolytic conditions.

**Fig. 2:**
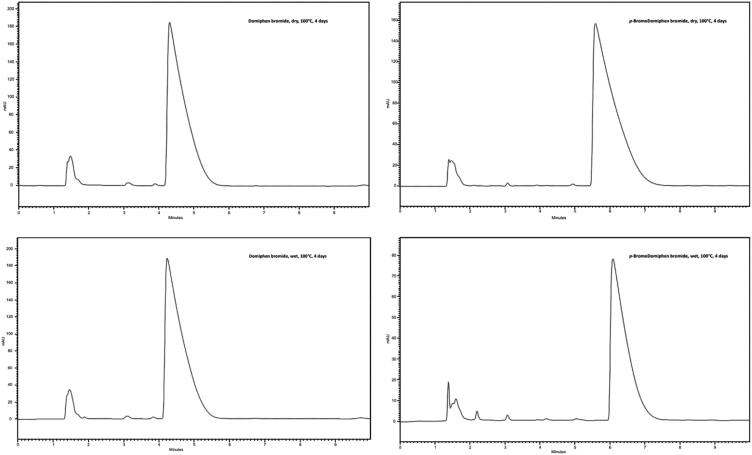
HPLC analyses of domiphen bromide and p-bromodomiphen bromide stressed under wet and dry thermal conditions.

All the chromatograms obtained are reported in Fig. 1, Fig. 2.

## 2 Experimental design, materials, and methods

The stress conditions were set as recommended by ICH and reported in [1].

Three samples of 100 mg each of domiphen bromide were respectively dissolved in 2 M HCl at 80 °C for 24 h, in 1 M NaOH at 80 °C and in 2 mL HPLC grade water at 100 °C for 4 days (oven).

Three samples of 100 mg each of *p-*bromo substituted domiphen bromide were, respectively, dissolved in 2 M HCl at 80 °C for 24 h, in 1 M NaOH at 80 °C and in 2 mL HPLC grade water at 100 °C for 4 days (oven).

For the dry thermal analyses, samples of 100 mg each of both domiphen bromide and *p-*bromo substituted domiphen bromide were kept at 100 °C for 4 days in oven.

All the up mentioned samples were analyzed by HPLC and the obtained chromatograms are reported below. Mobile phase A, and mobile phase B, for HPLC and LC/MS analyses were acetonitrile, and 3.0 mM ammonium formate (pH=4), respectively. The latter was prepared by dissolving ammonium formate (3.0 mM) in HPLC-grade water. The resulting solution was buffered to pH=4 by formic acid. An elution gradient was applied as reported below. Column temperature was fixed at 40 °C and chromatograms were extracted at 220 nm.

**Table:** 

**Time**	**% Eluent A**	**% Eluent B**	**Flow (mL/min)**
0	65	35	1.0
10	65	35	1.0
12	90	10	2.0
18	90	10	2.0
20	65	35	1.0
25	65	35	1.0
