# Supplementary material for: Data on thermal and hydrolytic stability of both domiphen bromide and para-bromodomiphen bromide
Source: Data Brief. 2018 Aug 31;20:1363–6. doi: 10.1016/j.dib.2018.08.152 (PMC6146566; doi:10.1016/j.dib.2018.08.152)
Supplement: Supplementary file 1 — Supporting information [file mmc1.docx]

Conflict of Interest

L. Fumagalli*,  G. Vistoli, M. Carini, C. Picozzi are inventors of a patent application focused on deriva- tives of p-bromo domiphen bromide as new antimicrobial agents.
